# A case report of non‐syndromic colonic ganglioneuroma in a patient with juvenile polyposis

**DOI:** 10.1002/jpr3.70223

**Published:** 2026-07-27

**Authors:** Holly Coffey, Kshitij Arora, Matthew J. Giefer

**Affiliations:** ^1^ The University of Queensland Ochsner Clinical School New Orleans Louisiana USA; ^2^ Department of Pathology LSU Health Shreveport Shreveport Louisiana USA

**Keywords:** endoscopic surveillance, MLH3 and MSH3 genes, polyps, sporadic

## Abstract

Colonic ganglioneuromas in children are rare, particularly without associated hereditary syndromes like multiple endocrine neoplasia (MEN2B), neurofibromatosis type 1(NF1), or Phosphatase and tensin homolog hamartoma tumor syndrome (PHTS). We report a 12‐year‐old male with a history of juvenile polyposis syndrome (JPS) and segmental colonic resection for high distal disease burden. During surveillance colonoscopy, a solitary ascending colon polyp was retrieved and found to be a ganglioneuroma. Genetic testing showed gl‐9 family hypoxia inducible factor 1 and succinate dehydrogenase complex flavoprotein subunit A variants in the patient and MutL homolog 3 and MutS homolog 3 variants in both the patient and his mother. All genes discovered were classified as variants of uncertain significance. There was no genetic or clinical evidence of MEN2B, NF1, or PHTS. This case highlights that JPS is a heterogenous disorder and may involve increased risk of developing unique polyp subtypes which are not typically associated with that condition. Current JPS recommendations suggest that surveillance colonoscopy may be performed every 3 years which may be insufficient in situations involving novel mutations or unknown JPS genetics.

## 1 INTRODUCTION

Colonic ganglioneuromas in children are rare. Patients with hereditary syndromes such as Multiple Endocrine Neoplasia (MEN2B), neurofibromatosis type 1 (NF1), or phosphatase and tensin homolog hamartoma tumor syndrome (PHTS) may present with multiple ganglioneuromas.^1^, ^2^ These syndromes typically present with multiple mucosal ganglioneuromas and additional characteristic features, such as pheochromocytomas, and medullary thyroid carcinomas.^1^, ^2^ Isolated cases of colonic ganglioneuromas in patients with juvenile polyposis syndrome (JPS) or in those without any hereditary syndromes are rare.^3^

## 2 CASE REPORT

**Figure 1:**
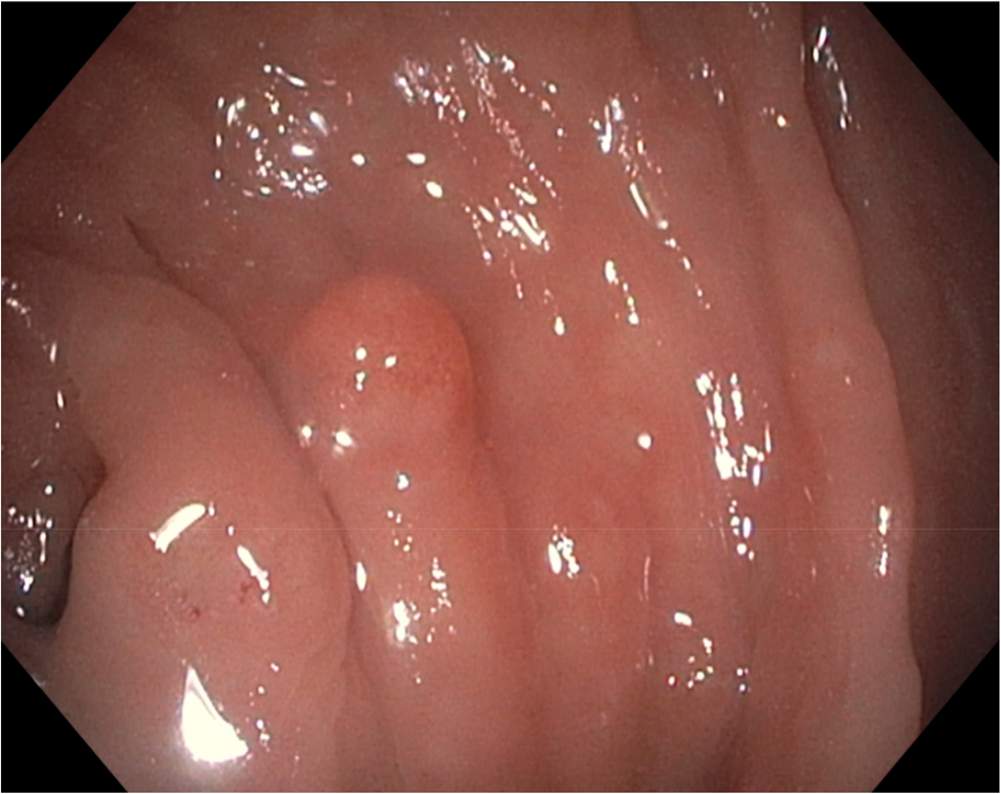
A ~5 mm ganglioneuroma in the proximal ascending colon.

**Figure 2:**
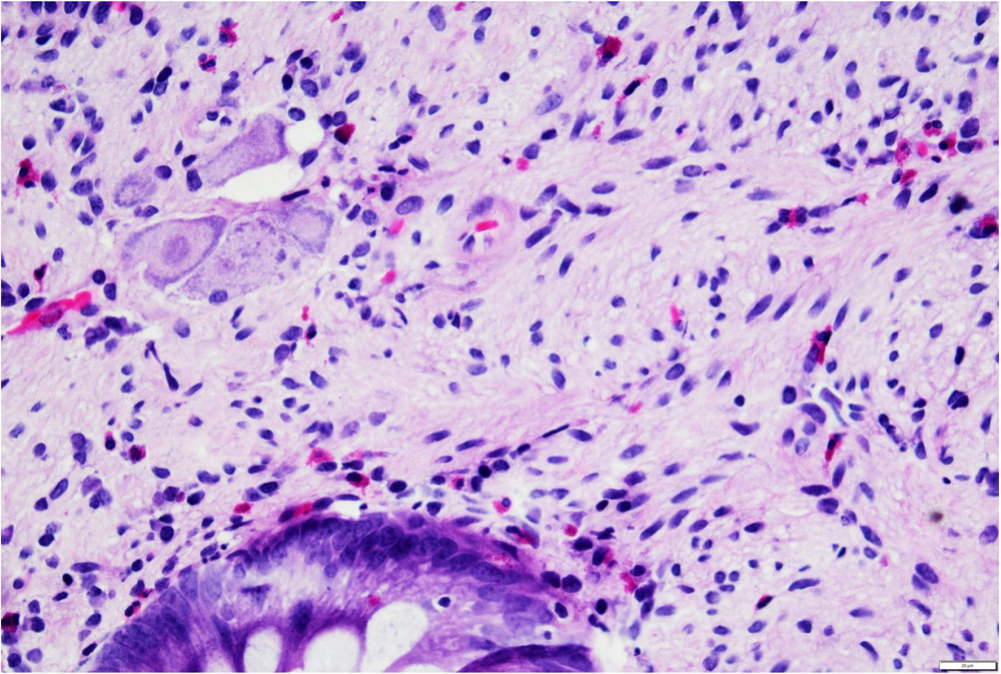
The lamina propria exhibits expansion with cytologically bland, spindled mesenchymal cells (SOX 10‐positive Schwann cells) that show no atypia or mitotic figures.

**Figure 3:**
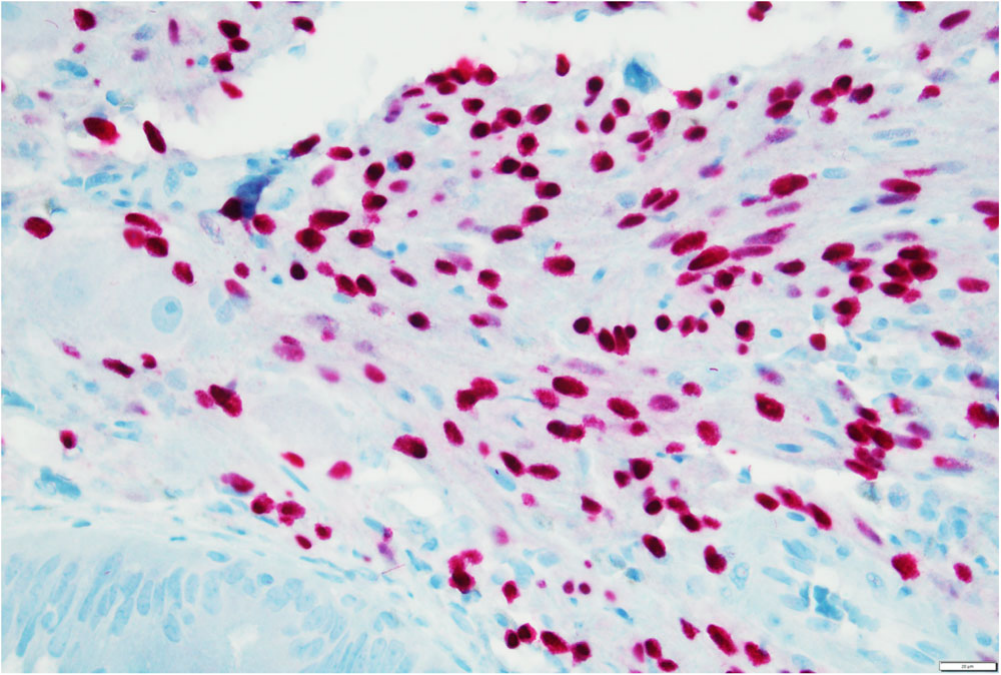
Additionally, ganglion cells are present (Hematoxylin & Eosin staining and SOX 10 immunohistochemistry at ×400 magnification).

A 2‐year‐old male with autism spectrum disorder and epilepsy presented with hematochezia. Index colonoscopy demonstrated three juvenile polyps. About 3 years later, four additional juvenile polyps, and one large (>15 cm) polyp were found on surveillance colonoscopy (according to abstracted records from another institution). A left hemi‐colectomy was performed at age five as the largest polyp could not be resected endoscopically. Post‐surgical surveillance colonoscopies 2 and 3 years later showed no additional polyps. About 4 years after surgery, at 9 years of age, an unspecified number of additional juvenile polyps were found and resected in the distal remaining colon. Two years later, a 5 mm semi‐pedunculated polyp was removed from the ascending colon (Figure 1), and histology revealed a ganglioneuroma (Figures 2 and 3). Follow‐up colonoscopy a year later confirmed complete excision of the ganglioneuroma and showed no additional polyps.

Initial genetic testing after the diagnosis of JPS identified:

MutL homolog 3 (MLH3) c. 1939C > T(pArg647Cys) (heterozygous)

MutS homolog 3 (MSH3) c.1571 A > C (p.Asn524Thr) (heterozygous)

Both the MLH3 and MSH3 mutations were identified in his mother.

Subsequent testing, following the ganglioneuroma resection identified:

Egl‐9 family hypoxia inducible factor 1 (EGLN1) c.493 C > A (p.Pro165Thr) (heterozygous)

Succinate dehydrogenase complex flavoprotein subunit A (SDHA) c.1424 G > A (p.Cys475Tyr) (heterozygous)

All four identified genes were classified as a variant of uncertain significance (VUS) with insufficient evidence to determine whether these variants are benign or pathogenic.

VUS represent alterations in a gene that have not been seen with sufficient frequency to know that they do or do not contribute to a specific cause of the disorder in question.

No mutations in genes commonly associated with JPS were identified including BMPR1A and SMAD4. No mutations consistent with MEN2B, NF1, PHTS, or other syndromic polyposis conditions were identified, and there was no clinical evidence to suggest a related syndrome.

## 3 DISCUSSION

Colonic ganglioneuromas are an uncommon gastrointestinal finding, particularly in children, and are typically associated with inherited conditions such as MEN2B, NF1, and PHTS.^1^, ^2^ Non‐syndromic ganglioneuromas have been described and may present with intussusception or intractable diarrhea due to enterochromaffin cell secretion of serotonin and other neuropeptides.^3^, ^4^, ^5^

Our case describing a ganglioneuroma in a patient with JPS and heterozygous MLH3 and MSH3 VUS suggests that these mismatch repair genes may be responsible for a unique JPS phenotype. MLH3 and MSH3 variants, are considered minor mismatch repair genes with less clearly defined contributions to cancer risk compared to the major mismatch repair genes.^6^ Biallelic pathogenic variants of MSH3 have been associated with an autosomal recessive adenomatous polyposis syndrome, and biallelic MLH3 has been implicated in similar polyposis phenotypes.^6^ The heterozygous VUS identified in this patient raises the possibility that less well‐characterized mismatch repair pathway genes may contribute to atypical polyposis phenotypes, or increased colorectal cancer risk, and their role in JPS and ganglioneuroma formation remains unclear. The EGLN1 and SDHA genes have not been linked to increased risk of polyps or other hereditary syndromes.^7^

A clinical diagnosis of JPS is often followed by genetic testing for identification of a pathogenic germline variant in SMAD4 or BMPR1A genes.^8^ Genetic testing may aid in confirming the diagnosis; however, approximately 40% of patients will not have pathogenic variants in their genes. This patient's phenotype and genetic testing results suggest that mismatch repair deficiency syndromes should also be considered.

Established clinical standards recommend initiating colonoscopic surveillance for JPS at ages 12–15 years with follow‐up exams every 1–3 years.^8^ JPS encompasses a wide spectrum of genetic abnormalities and phenotypic presentations and many patients who meet the clinical criteria for this condition lack an identifiable pathogenic variant or are found to have a VUS. Patients with poorly described genetic findings may be at either increased or decreased risk for severe phenotypic manifestations. This case underscores this heterogeneity and the need for clinicians to recognize the variable manifestations of JPS so surveillance and management can be individualized.

## 4 CONCLUSION

This case underscores the clinical and genetic heterogeneity of JPS and the limits of current genetic testing. Many patients who meet clinical criteria lack an identifiable pathogenic variant, and atypical polyps (such as ganglioneuroma as illustrated in this case) should raise suspicion for uncommon or novel genetic etiologies. Regardless of genetic findings—or when results are unknown—patients meeting clinical criteria for JPS should follow established, guideline‐recommended screening and surveillance protocols.

## CONFLICT OF INTEREST STATEMENT

The authors declare no conflicts of interest.

## ETHICS STATEMENT

Written informed consent for publication was obtained from the patient's parent/legal guardian.
